# Dietary Diversity and Inflammatory Diet Associated with All-Cause Mortality and Incidence and Mortality of Type 2 Diabetes: Two Prospective Cohort Studies

**DOI:** 10.3390/nu15092120

**Published:** 2023-04-28

**Authors:** Guzhengyue Zheng, Miao Cai, Huiling Liu, Rui Li, Zhengmin Qian, Steven W. Howard, Amy E. Keith, Shiyu Zhang, Xiaojie Wang, Junguo Zhang, Hualiang Lin, Junjie Hua

**Affiliations:** 1 Department of Epidemiology, School of Public Health, Sun Yat-sen University, Guangzhou 510080, Chinalinhualiang@mail.sysu.edu.cn (H.L.); 2 Food Inspection and Quarantine Center, Shenzhen Customs, Shenzhen 518016, China; 3 Department of Epidemiology and Biostatistics, College for Public Health & Social Justice, Saint Louis University, Saint Louis, MO 63104, USA; 4 Department of Health Services Administration, School of Health Professions, University of Alabama at Birmingham, Birmingham, AL 35233, USA

**Keywords:** mortality, dietary diversity score, energy-adjusted dietary inflammatory index, joint effects, type 2 diabetes

## Abstract

A higher dietary diversity score (DDS) and a lower energy-adjusted dietary inflammatory index (E-DII) may be associated with lower risks of type 2 diabetes (T2D) and mortality. This cohort study aimed to investigate the associations of DDS and E-DII with all-cause mortality, incidence of T2D, and mortality of T2D, as well as the joint effects of these two dietary factors. A total of 181,360 participants without all types of diabetes at baseline from the UK Biobank and 42,139 participants from the US NHANES were included. Cox proportional hazards models were used to assess the associations of DDS and E-DII with outcomes. In the UK Biobank data, 8338 deaths, 3416 incident T2D cases, and 353 T2D deaths occurred during a median follow-up of 12.5 years. In the US NHANES data, 6803 all-cause deaths and 248 T2D-specific deaths were recorded during a median follow-up of 9.6 years. We observed that higher DDS and lower E-DII were significantly associated with lower risks of total mortality and incident T2D. Compared with low DDS, the hazard ratios (HRs) and 95% confidence intervals (CIs) of high DDS were 0.69 (0.64, 0.74) for all-cause mortality, 0.79 (0.70, 0.88) for incident T2D in the UK Biobank, and 0.69 (0.61, 0.78) for all-cause mortality in the US NHANES. Compared with participants in tertile 3 of E-DII, those in tertile 1 had a lower risk of overall death [HR 0.86 (95% CI: 0.81, 0.91) in UK Biobank; 0.83 (0.77, 0.88) in US NHANES] and incident T2D [0.86 (0.79, 0.94)] in UK Biobank. No evidence was observed of the interactive effects of DDS and E-DII on either all-cause mortality or the incidence and mortality of T2D. There was no significant association found between any exposure and T2D mortality in this study. In conclusion, our results revealed that higher DDS and lower E-DII were associated with both total mortality and incident T2D in UK and US adults.

## 1. Introduction 

Diet is an important determinant of health [1]. In 2019, it was estimated that 8 million deaths and 188 million disability-adjusted life years (DALYs) were attributed to improper diet worldwide [2]. Consuming diverse foods is generally recommended by various dietary guidelines [3,4,5], and its health benefits, such as reducing inflammation, have been widely investigated [6]. These aspects of a healthy diet are of paramount importance in the prevention of type 2 diabetes (T2D) and other negative health outcomes [7,8,9]. Due to the substantial burden of T2D incidence and mortality [10,11], investigating the impact of dietary diversity and the inflammatory potential of diet on health is of substantial scientific value and public health interest.

Dietary diversity score (DDS) has been considered a useful indicator of nutritional adequacy and diet quality in previous surveys [12,13]. Some studies have investigated the associations between DDS and various health outcomes, including cognitive impairment, fracture, birth outcomes, diabetes, and mortality [14,15,16]. However, the findings regarding the relationship between DDS and health outcomes are contradictory. While some studies have shown a negative association between DDS and diabetes [9] and death [17], others found that higher dietary diversity is associated with obesity [18,19] or have found no significant association between DDS and diabetes [20]. Nevertheless, little is known about the potential causes associated with the heterogenous risk of T2D and death observed in epidemiological studies. In addition, observational studies that have suggested that dietary diversity has no significant effect on diabetes have some important limitations. These studies were limited in their ability to fully capture diet diversity, often using food frequency questionnaires as a means of assessment [20]. Additionally, some studies had small sample sizes [20] or were non-prospective in nature [18,19].

Dietary inflammatory index (DII) is associated with inflammatory biomarkers and systemic inflammation [21]. Several limited studies on the associations of DII with T2D and mortality have yielded conflicting results [22,23,24,25,26]. The studies mentioned above had significant limitations that hindered the generalizability of the findings. These limitations include being cross-sectional or case-control in design, having small sample sizes [27,28], using dietary inflammatory index calculation without adjustment for energy [29], or being comprised of women only [22]. It is crucial to investigate the effects of dietary diversity and inflammatory potential simultaneously on incident T2D and mortality. This is important because avoiding systematic inflammation may enhance the beneficial effects of a healthy diet on T2D and mortality [30]. To the best of our knowledge, no studies have reported on the combined associations of DDS and energy-adjusted dietary inflammatory index (E-DII) with risk of all-cause mortality, incidence, and mortality of T2D. 

To address these research gaps, we conducted this study based on two large-scale prospective cohorts of the UK Biobank and the US National Health and Nutrition Examination Survey (NHANES), with repeated measures of diet and long median follow-ups. The aim was to investigate the associations of DDS and E-DII with total mortality, incidence, and mortality of T2D, as well as their combined associations.

## 2. Materials and Methods

### 2.1. Study Population

The UK Biobank is a large prospective cohort of more than 0.5 million participants (aged 37–73 years) recruited from 22 UK assessment centers across Wales, Scotland, and England between 2006 and 2010 [31]. A wide range of health data was obtained via questionnaires, verbal interviews, and biological and physical measurements. Among the 502,461 adults, 211,025 participants completed at least one of the five rounds of 24-h dietary recall surveys (the time points of the five rounds of dietary surveys are shown in Supplemental Figure S1), which were conducted using the Oxford WebQ between 2009 and 2012 [32]. Participants who met certain criteria were excluded from this study. These criteria included missing or unbelievable dietary information (participants not consuming any food in the past day; *n* = 5888), withdrawal of consent for linkage (*n* = 16), implausible daily low caloric intakes (women: <600 kcal; men: <800 kcal) or high intakes (women: >3500 kcal; men: >4200 kcal) (*n* = 5781), missing data on key covariates (age, sex, race, residence, calorie intake from diet, and BMI; *n* = 8164), or occurrences of all types of diabetes at baseline (type 1 diabetes, type 2 diabetes, gestational diabetes, other types of diabetes; *n* = 9816). After exclusion, the final analyses included 181,360 individuals (Supplemental Figure S2).

The US NHANES study was designed to represent the population of the US using stratified four-stage probability random sampling. We used 11 cycles of the US NHANES (1999–2000 to 2019–2020) and linked them with the National Center for Health Statistics (NCHS) 2019 mortality file to create an initial large cohort. The mortality follow-up information and corresponding linked mortality files (LMF) were updated through 31 December 2019 [33]. The current analysis included 116,876 participants at baseline. We excluded participants who were aged < 20 years, pregnant, had missing death information (*n* = 61,424), had implausible daily low-calorie intakes (women: <600 kcal; men: <800 kcal) or high intakes (women: >3500 kcal; men: >4200 kcal) (*n* = 1927), those with non-reliable dietary recall records, or had missing information on other key covariates (age, sex, race, calorie intake from diet, and BMI; *n* = 8891), those with occurrences of all types of diabetes (*n* = 2495) at baseline. The final analyses included 42,139 participants (Supplemental Figure S3).

### 2.2. Assessment of DDS

In the UK Biobank, dietary information was assessed through five rounds of 24-h dietary recall surveys. In the US NHANES, dietary intake data were evaluated through one round of 24-h dietary recall interviews from 1999 to 2002 and two rounds of 24-h dietary recalls (two consecutive days) from 2003 to 2020. Notably, we calculated mean values for the number of repeated dietary assessments. Dietary diversity score (DDS), which was developed by Kant et al. [34], has been validated in other cohorts [14,15,16,35,36,37]. According to the United Nations' Food and Agriculture Organization food group classification guidance [38], we constructed DDS to assess dietary diversity based on five major food groups (eighteen subgroups): grains (whole grains, non-whole/refined grains), vegetables (dark green leafy, vitamin A-rich, starchy tubers, other), fruits (citrus, vitamin A-rich, other), meat and protein alternatives (red meat, fish and seafood, poultry, organ meat, eggs, legumes, and nuts), and milk products (yogurt, milk, cheese). The dietary diversity score increased for each food subcategory in which a participant consumed any food, but diverse foods consumed within the same subcategory were not calculated repeatedly. The DDS was equal to the sum of the points for all eighteen subgroups mentioned above. We considered that it was impossible for a person’s normal diet to consist of eating nothing for a day, so the total DDS ranged from 1 to 18. A higher DDS reflects a richer diet and is associated with meeting the needs of all essential nutrients. The same method was used to assess dietary diversity in two cohorts, albeit with slightly different food-specific items (Supplemental Table S1). 

### 2.3. Assessment of E-DII

A detailed description of the development and validation of the dietary inflammatory index (DII) has been previously described [21]. Briefly, over 6500 papers published from January 2008 to December 2010 were screened. Of those articles, qualified articles were scored to generate the scores reflecting inflammation for 45 dietary parameters (nutrients, individual foods, or bioactive components), which were elements of the dietary inflammatory index. These dietary components were associated with six anti- or pro-inflammatory biomarkers [tumor necrosis factor-α, interleukin (IL)-10, IL-6, IL-4, IL-1β, and *C*-reactive protein]. Based on its association with the inflammatory biomarkers, a value was allocated to each eligible article: −1, 0, or +1 for an anti-inflammatory, no effect, or pro-inflammatory, respectively. The value for each paper was weighted according to the type of research design, and component-specific inflammatory scores were assigned according to weighted scores. Based on an international dietary database consisting of 11 datasets from different populations around the world, the dietary data were further standardized. A participant’s dietary inflammatory index was calculated by multiplying the component-specific inflammatory scores with the standardized dietary data and then summing them together. To control for confounding and predict the effect of diet accurately, we used the nutrient density approach [39] to obtain the energy-adjusted dietary inflammatory index (E-DII), which was used in our statistical analyses. A lower E-DII score reflected a more anti-inflammatory diet, and vice versa.

There were some missing values for some dietary items of the 45 dietary components in the two cohorts, so we used dietary data for 28 dietary components both in the UK Biobank and US NHANES for the E-DII calculation. Notably, two of the 28 dietary components were different: trans fats in the UK Biobank and caffeine in the US NHANES (Supplemental Table S2). In the UK Biobank, for participants who had completed 24-h dietary recall questionnaires two or more times, we calculated the mean food or nutrient intake from their records. In the US NHANES, the amount of one specific food or nutrient intake was the direct intake of one individual at the time of the dietary survey during the period from 1999 to 2002 and was calculated by the mean of the two rounds of dietary intake from 2003 to 2020.

### 2.4. Outcome Ascertainment

The outcomes of this study were all-cause mortality, incidence from T2D, and mortality of T2D. These outcomes were ascertained in accordance with ICD-10 [the International Classification of Diseases (ICD), 10th revision] codes. In the UK Biobank data, those outcomes that occurred during follow-up were identified through linkage to the hospital admissions and death registries. The electronic data of the hospital admission and death registry contained primary and secondary care records across healthcare systems in Wales, Scotland, and England. Hospital admission and death data were available through 31 December 2021. Specifically, incident T2D was defined as insulin-dependent diabetes (E11). The mortality of T2D was defined as death directly after T2D diagnosis or death from diabetes complications. Diabetic complications included diabetic retinopathy, cerebrovascular disease, diabetic neuropathy, cardiovascular disease (CVD), peripheral vascular disease, diabetic nephropathy, T2D with coma, T2D with ketoacidosis, elevated blood sugar, other specified diabetes mellitus complications, and other unspecified multiple complications of diabetes. In US NHANES, all-cause and T2D mortality were obtained via the National Death Index through 31 December 2019.

### 2.5. Covariates

At baseline, the covariates were obtained through questionnaires that included sociodemographic factors [age (continuous), sex (male, female), race (white, non-white), household income (high, medium, and low), residence (urban, rural) (UK Biobank only)], alcohol consumption (g/week) (continuous), smoking status (never, previous, current), physical activity (continuous), calorie intake from the diet (kcal/day) (continuous), dietary supplement (yes/no), family history of diabetes (yes/no), and health condition [body mass index (BMI) (continuous), CVD (yes/no), cancer (yes/no), hypertension (yes/no), and high cholesterol (yes/no)]. Specifically, age was calculated from birth dates and baseline assessment dates. Physical activity was defined as the metabolic equivalent of a task in hours per week. BMI is calculated as weight (kg) divided by height^2^ (m^2^). Information on CVD, cancer, hypertension, and hyperlipidemia was obtained through hospital inpatient records and self-reported data at baseline.

### 2.6. Statistical Analysis

Descriptive statistics were used to describe basic characteristics by mean (standard deviation) or frequency (proportion). The baseline characteristics of participants were shown as three DDS categories and tertiles of E-DII, respectively. The Spearman correlation methods were conducted to diagnose correlations between variables (DDS, E-DII, and all covariates).

We grouped DDS into three groups according to practical implications for public health: low DDS (ranges 1–6), medium DDS (ranges 7–12), and high DDS (ranges 13–18). The reference group was set as the participants with low DDS. The E-DII was divided into tertiles, with the highest tertile (tertile 3 of the E-DII) as the reference.

Cox proportional hazards regression models were performed to investigate the associations of DDS and E-DII with all-cause mortality, the incidence of T2D, and the mortality of T2D. Notably, we calculated the person-years from the date of returning the baseline questionnaire to the date of death, T2D diagnosis, the endpoint of the follow-up period, or loss to follow-up, whichever came first. We selected a priori potential confounders for adjustment in multivariable regression models based on knowledge of clinically relevant factors or their relationships with dietary exposures and outcomes [40,41]. Models were adjusted for age, sex, race, household income, residence (UK Biobank only), family history of diabetes (included in analyses when using incidence or mortality of T2D as outcomes), smoking status, alcohol consumption, physical activity, BMI, and total calorie intake from the diet (model 1), plus dietary supplement, CVD, cancer, hypertension, and hyperlipidemia (model 2). A linear trend was tested across three levels of DDS and tertiles of E-DII by assigning the midpoint value to each group of DDS and each tertile of E-DII and considering these two variables as continuous variables in their respective models. Furthermore, we explored the shape of the associations between DDS (or E-DII) and outcomes by fitting a restricted cubic spline function with four knots (Q_5_, Q_35_, Q_65_, and Q_95_).

The potential joint effects of DDS and E-DII were assessed in both multiplicative and additive interactive models. Interaction on a multiplicative or additive scale suggests that the joint effect of the two exposures is smaller/larger than the product or sum of their independent effects [42]. Multiplicative interaction was investigated by adding a product term between DDS and E-DII to model 2 and then tested by comparing the −2 log likelihood values with and without the cross-product interaction term [43]. For additive interaction, we generated a term with nine categories (3 × 3) for three DDS categories (low, medium, and high) and tertiles of E-DII. We further calculated the relative excess risk due to interaction (RERI) and their 95% CIs in model 2 to estimate the additive interaction. We set participants with low DDS and tertile 3 of E-DII as the combined reference subgroup. A RERI of 0, less than 0, and more than 0 indicated no, negative, and positive additive interactions, respectively.

To test potential heterogenicity across different subgroups, stratified analyses were further conducted by age group (<55 and ≥55 years), sex (female and male), and BMI (<25 and ≥25 kg/m^2^). 

We conducted several sets of sensitivity analyses. First, we excluded participants who had a T2D or mortality occurrence within 2 years after finishing their last 24-h dietary records, considering reverse causality. Second, we excluded participants aged 20–40 years in the US NHANES to alleviate concerns that dietary habits are prone to change, that the risk of illness or mortality due to diet is relatively lower in younger individuals, and to be consistent with the age distribution in the UK Biobank. Third, in model 2 in the UK Biobank study, we calculated DDS using food frequency questionnaire (FFQ) data [more information on FFQ and the process of calculating DDS based on it can be found in the Supplemental Material (Supplemental Methods)] and further assessed the associations of DDS (based on FFQ) with the chosen outcomes. 

A *p*-value smaller than 0.05 was considered statistically significant. All statistical analyses were conducted using R software (version 4.2.1, Auckland, New Zealand).

## 3. Results

### 3.1. Participant Characteristics

Compared with the final sample (181,360 participants in the UK Biobank and 42,139 participants in the US NHANES) in the analyses, all participants at baseline (211,025 in the UK Biobank and 116,879 in the US NHANES) tended to be older, male, non-white, live in cities, smoke more cigarettes, drink more alcohol, and be more inactive. For the final UK Biobank sample, the mean (SD) baseline age was 55.9 (7.9) years, 101,298 (55.9%) were female, and 174,392 (96.2%) were white people. In US NHANES, the mean (SD) baseline age was 50.1 (18.1) years, 22,384 (53.1%) were female, and 19,472 (46.2%) were white people. No statistically significant correlation was identified between the independent variables, suggesting that collinearity was not a concern.

The range (median) of the DDS was 1–18 (10) in the UK Biobank and 1–18 (12) in the US NHANES (Table 1). In the two cohorts, participants with higher DDS tended to be older, female, white, have a higher household income, consume more energy from the diet, smoke cigarettes and drink alcohol less, have a lower BMI, and have a higher dietary supplement intake. E-DII ranged from −5.44 (the anti-inflammatory potential was the strongest) to 4.65 (the anti-inflammatory potential was the weakest) in the UK Biobank and from −5.28 (the anti-inflammatory potential was the strongest) to 5.79 (the anti-inflammatory potential was the weakest) in the US NHANES. In the two cohorts, compared to adults in tertile 3 of E-DII (highest inflammation), those in tertile 1 (lowest inflammation) were more likely to be older, male, white, have a higher household income, be non-smokers, have a higher caloric intake, drink more alcohol, and have a lower BMI.

### 3.2. Associations of DDS with Outcomes

In the UK Biobank data, 8338 total deaths, 3416 incident T2D cases, and 353 deaths of T2D occurred over 2,255,204 person-years (with a median follow-up of 12.5 years). In the US NHANES data, 6803 overall deaths and 248 deaths from T2D were documented over 5,016,292 person-years (with a median follow-up of 9.6 years). Table 2 shows the associations between DDS and overall death, the incidence of T2D, and mortality of T2D in participants from the UK Biobank and US NHANES. We observed an inverse association between DDS and the risk of overall death and incident T2D (Table 2). After accounting for multiple potential confounding factors in model 2, each 1-point increment in the DDS was associated with a 3.9% (95% CI: 3.2, 4.6) lower risk of overall death, a 2.6% (1.5, 3.7) incident T2D in the UK Biobank, and a 4.4% (3.4, 5.4) all-cause mortality in the US NHANES. The HRs (95 CIs) when participants of high DDS were compared with participants of low DDS (13–18 vs. 1–6) were 0.69 (0.64, 0.74; *p*_trend_ < 0.001) for total mortality, 0.79 (0.70, 0.88; *p*_trend_ < 0.001) for incident T2D in UK Biobank, and 0.69 (0.61, 0.78; *p*_trend_ < 0.001) for overall death in US NHANES. Similar results were observed in Model 1. The associations were no longer significant between DDS and T2D mortality in the two models. Results were not fundamentally changed in any of the sensitivity analyses (Supplemental Tables S3, S6 and S8).

Additionally, the dose-response relationships of DDS with health outcomes in the UK Biobank and US NHANES were further assessed by restricted cubic spline models. Overall, a linear dose-response relationship was observed between DDS and total mortality (*p* for non-linearity = 0.32 in the UK Biobank and 0.19 in the US NHANES) as well as incident T2D (*p* for non-linearity = 0.18 in the UK Biobank). However, a significant non-linear association of DDS with T2D mortality (*p* for non-linearity < 0.001) was observed in the participants from the two cohorts. The observed curves suggested that HRs for risk of overall death and incident T2D decreased with the increase in DDS (Supplemental Figure S4). 

### 3.3. Associations of E-DII with Outcomes

In Table 3, we examined the associations of E-DII with all-cause mortality, incident T2D, and T2D mortality in participants from the UK Biobank and US NHANES. After adjusting for potential covariates in model 2, we found that a decrease in 1-point in the E-DII was associated with a 3.3% (95% CI: 1.1, 5.5) lower risk of incident T2D, a 3.8% (2.4, 5.2) lower risk of overall death in the UK Biobank cohort, and a 5.0% (3.5, 6.5) lower risk of overall death in the US NHANES cohort. Additionally, we also assessed the relationship between tertiles of the E-DII score and all-cause mortality as well as the incidence and mortality of T2D. Multivariable-adjusted analysis in model 2 showed that the HR (95% CI) of incident T2D decreased by 14% among the participants in tertile 1 of E-DII [0.86 (0.79, 0.94); *p*_trend_ = 0.032], and the HR of all-cause mortality decreased by 14% among the individuals in tertile 1 of E-DII [0.86 (0.81, 0.91); *p*_trend_ < 0.001] in the UK Biobank data compared with participants in tertile 3 of E-DII. Similarly, adults from the US NHANES data in tertile 1 of E-DII had a 17% lower HR of all-cause death than those in tertile 3 of E-DII [0.83 (0.77, 0.88); *p*_trend_ < 0.001). The associations between E-DII and T2D mortality were not significant in the two models. The results of all sensitivity analyses were not substantially altered (Supplemental Tables S4 and S6). The assumption of linearity between E-DII and the risk of total mortality (with respective *p*-values of 0.42 in UK Biobank and 0.39 in US NHANES), as well as incident T2D (with a *p*-value for the non-linear association of 0.56 in the UK Biobank), was confirmed by the restricted cubic spline. However, a significant non-linear association was observed between E-DII and T2D mortality (with a *p*-value for non-linearity < 0.001 in both cohorts) (Supplemental Figure S5).

### 3.4. Combined Effects of DDS and E-DII with Outcomes

The combined associations of DDS and E-DII on all-cause mortality, the incidence of T2D, and mortality of T2D are shown in Table 4. Participants with higher DDS and lower E-DII generally had a lower risk of total mortality and incident T2D. Using the participants with low DDS and tertile 3 of E-DII as the reference, those with high DDS and tertile 1 of E-DII had the lowest HRs (95% CIs) of overall death [0.61 (0.55, 0.68) in UK Biobank, 0.58 (0.49, 0.68) in US NHANES] and incident T2D [0.75 (0.64, 0.89)] in UK Biobank. Tests for multiplicative interactions were not significant for all outcomes in both cohorts (all *p*_interaction_ > 0.05). We observed similar results on an additive scale. Results were not fundamentally altered in the sensitivity analyses (Supplemental Tables S5 and S7).

### 3.5. Stratified Analyses

Supplemental Tables S9 and S10 show results stratified by age group (<55 and ≥55 years), sex, and BMI (<25 and ≥25 kg/m^2^). Most of the statistically significant interactions observed lacked major implications. Stratified analyses indicated that the association between DDS (or E-DII) and the risk of overall death and the incidence and mortality of T2D were not modified by sex, age, or BMI (*p*_interaction_ > 0.05).

## 4. Discussion

This was the first study to simultaneously assess the independent and joint effects of dietary diversity and dietary inflammatory potential on total mortality, the incidence of T2D, and mortality from T2D in UK and US adults. In these two large cohorts, both higher DDS and lower E-DII were associated with a lower risk of overall death and incident T2D. Little evidence of combined associations between DDS and E-DII on outcomes was observed on either multiplicative or additive scales.

### 4.1. Comparison with Previous Studies and Biological Mechanisms

The inverse association between adherence to a higher diversity diet and a lower risk of overall death and incident T2D observed in the UK Biobank and US NHANES data sets is generally in line with previous studies. For instance, similar to our study, one study based on the EPIC-Norfolk cohort of 23,238 participants reported that higher DDS reflecting more dietary diversity could decrease the risk of incident T2D [9]. In addition to reducing the risk of T2D, higher dietary diversity was also reported to be associated with reduced risks of other outcomes, including cognitive impairment [14], fracture [15], anxiety [44], and mortality [35]. However, in a multi-ethnic study of atherosclerosis where diet information was assessed among 5160 white, Hispanic, Black, and Chinese individuals, no significant association was observed between dietary diversity and diabetes [20]. Differences in dietary measurement and assessment methods for the dietary diversity indicator may explain this heterogeneity. We obtained dietary information from repeated 24-h dietary recall surveys and further assessed dietary diversity based on five major food groups (eighteen subgroups). This differed from the methods used by Otto et al. [20], which involved using dietary data based on a food frequency questionnaire survey to assess three aspects (count, evenness, and dissimilarity) of dietary diversity. The observed heterogeneity might also have been due to differences in population characteristics, location, and study period.

The mechanisms underlying the associations of dietary diversity with all-cause mortality and incident T2D are unclear. The health benefits of high dietary diversity may be partly attributed to the microbial community structure and α-diversity of the human gut microbiome [45]. Greater diversity of food consumption may also play key roles in reducing the risk of T2D incidence and death by providing sufficient dietary fiber, plant protein, trace elements, vitamin A, vitamin B_6_, vitamin B_12_, vitamin C, vitamin D, vitamin E, folic acid, amino acids, long-chain omega-3 fatty acids, phytochemicals, and bioactive components [46,47,48,49,50].

The inverse association between an anti-inflammatory diet and an increased risk of T2D and death was consistent with the literature [25,51]. A study of 1024 adults in two Dutch cohorts found that a lower E-DII was associated with a lower prevalence of diabetes [52]. Mena et al. reported that adhering to an anti-inflammatory diet might reduce the risk of all-cause mortality [23]. However, controversial results remain. For example, there was no association found between DII and fasting glucose in a study of Croatian workers [53]. Evertine et al. found that there was no association with all-cause mortality in colorectal cancer patients [51]. Differences in health outcomes and study populations may explain the heterogeneity. For example, while the focus of this study was primarily on T2D incidence and mortality, the study by Kendel et al. concentrated on fasting glucose, and the findings of Evertine et al. were based on people with CRC.

The DDS and E-DII were weakly correlated (the Pearson correlation coefficient was −0.29 in the UK Biobank study and −0.23 in the US NHANES study). The weak correlations between E-DII and DDS can be explained by the fact that they share few dietary components. E-DII places a greater emphasis on unique foods associated with inflammation [21]. For instance, it includes many anti-inflammatory compounds such as fiber, beta-carotene, vitamins, *n*-3 fatty acids, *n*-6 fatty acids, and flavonoids while limiting the intake of the proinflammatory components such as energy, total fat, cholesterol, and trans-fat [54]. In this analysis of the joint effects of DDS and E-DII on total mortality and incidence and mortality of T2D within the same tertile of E-DII (or DDS group), participants adhering to higher DDS (or lower E-DII) seemed to have a lower HR than those with lower DDS (or higher E-DII). However, the observed differences were too slight to be statistically significant, suggesting little evidence of combined associations between DDS and E-DII with outcomes. There are two possible reasons why we found little evidence of combined associations of DDS and E-DII with outcomes. Firstly, while both a higher diversity diet and an anti-inflammatory diet may contribute to a lower risk of insulin resistance, metabolic syndrome, immune function, change of hormonal levels, and inflammation [55,56,57], the benefits from a higher dietary diversity may outweigh the protective effect of an anti-inflammatory diet. One possible reason is that the DDS and E-DII may have similar biological mechanisms in terms of their health effects (via microbiome) [58]. Additionally, there may be less of an anti-inflammatory effect for tertile 1 of E-DII in the low DDS group compared with the same E-DII tertile in the high-DDS and medium DDS. For example, the HRs of tertile 2 and tertile 1 of E-DII with overall death were 0.87 (95% CI: 0.75, 1.00) and 0.90 (0.72, 1.11) in the UK Biobank study, respectively. The possible reason was that anti-inflammatory dietary components did not maximize their anti-inflammatory effects since people with low dietary diversity and tertile 1 of E-DII might consume too few types of food.

### 4.2. Strengths and Limitations

This study has several strengths. To our knowledge, it was the first study to simultaneously investigate the combined associations of dietary diversity and an inflammatory diet with all-cause mortality and the incidence and mortality of T2D. The major strengths of our analysis included large sample size, repeated assessments of dietary information, a long follow-up with many health outcomes, and the examination of combined DDS and E-DII. The consistency of the results from the two cohorts suggests that our findings are not likely to be due to chance. The large sample size not only enabled us to conduct the stratified and joint effect analyses but also ensured that the results were more reliable.

This study also has several limitations. First, information on diet was self-reported, which may introduce information bias into the study. However, we used data from five rounds of 24-h dietary recall records in the UK Biobank and two rounds of 24-h dietary recall records in the US NHANES to reduce the possibility of measurement errors. In addition, we calculated DDS using FFQ information and further assessed the association of this DDS with outcomes repeatedly. Second, DDS scores are derived from whether individuals consumed those 18 food groups, regardless of the amount of food consumed, which may not reflect the real dietary diversity status. Future studies that include the amount of dietary intake would be preferred. Third, the follow-up period is not long enough. In addition, the long course of diabetes and the fact that diabetes patients generally do not die directly from diabetes may cause the sample size of diabetes deaths to be small, limiting the analyses of this study. This may explain some of the observed non-significant associations between exposures and T2D mortality. However, we defined T2D mortality as diabetes patients who died from diabetes directly or from diabetes complications during the follow-up period. Additionally, due to the categorization of race as either white or non-white, we were unable to conduct cross-racial comparisons to assess whether these effects differ across more specific racial categories [59]. Finally, despite adjusting for key covariates based on the directed acyclic graph and previous studies, the possibility of residual confounding cannot be ruled out, and causal inference cannot be made due to the shortcomings of observational studies.

## 5. Conclusions

In conclusion, we found that higher DDS and lower E-DII were independently associated with a lower risk of overall death and incident T2D. Our findings did not provide support for the interaction effects between DDS and E-DII on outcomes.

## Figures and Tables

**Table 1:** Baseline characteristics of participants from the UK Biobank and US NHANES according to DDS and E-DII subgroups ^a^.

Variables	Overall	DDS Categories			E-DII		
		Low (1–6)	Medium (7–12)	High (13–18)	Tertile 1 ^b^	Tertile 2 ^b^	Tertile 3 ^b^
**UK Biobank**							
No of participants	181,360	22,542 (12.4)	111,231 (61.3)	47,587 (26.2)	60,454 (33.3)	60,453 (33.3)	60,453 (33.3)
Age [mean (SD)]	56.0 (8.0)	53.9 (8.1)	55.9 (8.0)	57.1 (7.5)	56.6 (7.9)	56.0 (7.9)	55.2 (8.0)
Female	101,298 (55.9)	10,866 (48.2)	61,148 (55.0)	29,284 (61.5)	29,706 (49.1)	34,382 (56.9)	37,210 (61.6)
Race Non-white	174,392 (96.2)	1451 (6.4)	4245 (3.8)	1272 (2.7)	1960 (3.2)	1905 (3.2)	3103 (5.1)
Residence Rural	29,549 (16.3)	3161 (14.0)	18,128 (16.3)	8260 (17.4)	10,125 (16.7)	10,071 (16.7)	9353 (15.5)
Household income							
High	52,849 (29.1)	5836 (25.9)	32,372 (29.1)	14,641 (30.8)	17,297 (28.6)	18,302 (30.3)	17,250 (28.5)
Medium	85,945 (47.4)	10,342 (45.9)	52,744 (47.4)	22,859 (48.0)	29,281 (48.4)	28,608 (47.3)	28,056 (46.4)
Low	24,076 (13.3)	3751 (16.6)	14,797 (13.3)	5530 (11.6)	8125 (13.4)	7487 (12.4)	8464 (14.0)
Smoking status							
Never	103,480 (57.1)	11,935 (52.9)	63,299 (56.9)	28,246 (59.4)	34,833 (57.6)	34,784 (57.5)	33,863 (56.0)
Previous	63,482 (35.0)	7438 (33.0)	39,068 (35.1)	16,978 (35.7)	21,715 (35.9)	21,355 (35.3)	20,412 (33.8)
Current	14,006 (7.7)	3092 (13.7)	8618 (7.7)	2296 (4.8)	3790 (6.3)	4200 (6.9)	6016 (10.0)
Alcohol consumption (g/week), mean (SD)	110.3 (96.5)	126.06 (119.5)	110.66 (96.2)	101.97 (83.2)	115.3 (100.5)	110.1 (95.4)	105.6 (93.4)
PA MET(hour/week), mean (SD)	41.7 (37.6)	42.2 (40.7)	41.5 (37.6)	41.8 (36.1)	45.3 (40.0)	40.7 (36.4)	39.3 (36.3)
BMI (kg/m^2^), mean (SD)	26.7 (4.4)	27.5 (4.7)	26.7 (4.4)	26.3 (4.3)	26.6 (4.4)	26.6 (4.4)	26.9 (4.6)
Energy intake (kcal/day), mean (SD)	2071.5 (613.4)	1865.9 (631.6)	2076.0 (607.9)	2158.7 (594.4)	2447.9 (594.3)	2068.3 (512.5)	1698.5 (481.5)
Dietary supplement	86,862 (47.9)	9427 (41.8)	52,782 (47.5)	24,655 (51.8)	30,976 (51.2)	28,944 (47.9)	26,942 (44.6)
Family history of diabetes	37,247 (20.5)	4925 (21.8)	22,690 (20.4)	9632 (20.2)	12,062 (20.0)	12,450 (20.6)	12,735 (21.1)
Comorbidities							
CVD	6602 (3.6)	982 (4.4)	4078 (3.7)	1542 (3.2)	2296 (3.8)	2143 (3.5)	2163 (3.6)
Cancer	15,626 (8.6)	1700 (7.5)	9461 (8.5)	4465 (9.4)	5307 (8.8)	5207 (8.6)	5112 (8.5)
Hypertension	75,120 (41.4)	9248 (41.0)	45,869 (41.2)	20,003 (42.0)	25,795 (42.7)	24,957 (41.3)	24,368 (40.3)
Hyperlipidemia	24,899 (13.7)	3105 (13.8)	15,337 (13.8)	6457 (13.6)	8670 (14.3)	8286 (13.7)	7943 (13.1)
Total deaths	8338 (4.6)	1218 (5.4)	5201 (4.7)	1919 (4.0)	2814 (4.7)	2658 (4.4)	2866 (4.7)
Incident T2D cases	3416 (1.9)	577 (2.6)	2077 (1.9)	762 (1.6)	1159 (1.9)	1033 (1.7)	1224 (2.0)
T2D deaths	353 (0.2)	54 (0.2)	208 (0.2)	91 (0.2)	122 (0.2)	95 (0.2)	136 (0.2)
**US NHANES**							
No of participants	42,139	1171 (2.8)	23,638 (56.1)	17,330 (41.1)	14,047 (33.3)	14,046 (33.3)	14,046 (33.3)
Age [mean (SD)]	50.1 (18.1)	47.3 (17.8)	49.5 (18.2)	51.2 (17.9)	50.2 (17.6)	49.9 (18.1)	50.3 (18.6)
Female	22,384 (53.1)	497 (42.4)	11,999 (50.8)	9888 (57.1)	6047 (42.6)	7456 (53.2)	8881 (63.7)
Race White	19,472 (46.2)	476 (40.6)	10,789 (45.6)	8207 (47.4)	6841 (48.2)	6404 (45.7)	6227 (44.7)
Household income							
High	9656 (22.9)	129 (11.0)	4304 (18.2)	5223 (30.1)	4175 (29.4)	3115 (22.2)	2366 (17.0)
Medium	21,343 (50.6)	555 (47.4)	12,179 (51.5)	8609 (49.7)	6901 (48.6)	7162 (51.1)	7280 (52.2)
Low	8589 (20.4)	364 (31.1)	5528 (23.4)	2697 (15.6)	2255 (15.9)	2841 (20.3)	3493 (25.1)
Smoking status							
Never	22,990 (54.6)	467 (39.9)	12,069 (51.1)	10,454 (60.3)	8011 (56.5)	7684 (54.9)	7295 (52.3)
Previous	10,814 (25.7)	224 (19.1)	5874 (24.8)	4716 (27.2)	4075 (28.7)	3557 (25.4)	3182 (22.8)
Current	8306 (19.7)	475 (40.6)	5677 (24.0)	2154 (12.4)	2090 (14.7)	2760 (19.7)	3456 (24.8)
Alcohol consumption (g/week), mean (SD)	95.3 (44.5)	95.1 (69.7)	69.7 (39.8)	60.4 (28.1)	60.8 (32.2)	61.3 (35.0)	54.3 (30.3)
PA MET(hour/week), mean (SD)	54.3 (77.4)	46.6 (65.1)	55.8 (80.7)	52.8 (73.3)	57.5(82.0)	55.6 (77.7)	59.4 (83.3)
BMI (kg/m^2^), mean (SD)	29.1 (6.7)	28.7 (6.7)	29.2 (6.8)	28.9 (6.6)	28.4 (6.2)	29.2 (6.7)	29.6 (7.2)
Energy intake (kcal/day), mean (SD)	1988.1 (716.3)	1585.8 (723.6)	1910.8 (719.4)	2120.7 (685.3)	2370.1 (706.0)	1976.6 (638.5)	1610.8 (585.5)
Dietary supplement	13,529 (32.1)	89 (7.6)	6020 (25.5)	7420 (42.8)	5122 (36.1)	4215 (30.1)	4192 (30.1)
Comorbidities							
CVD	4805 (11.4)	140 (12.0)	2853 (12.1)	1812 (10.5)	1360 (9.6)	1581 (11.3)	1864 (13.4)
Cancer	3413 (8.1)	97 (8.3)	2057 (8.7)	1259 (7.3)	1054 (7.5)	1110 (7.9)	1249 (8.9)
Hypertension	18,387 (43.6)	514 (43.9)	10,423 (44.1)	7450 (43.0)	5835 (41.1)	6109 (43.6)	6443 (46.2)
Hyperlipidemia	12,304 (29.2)	359 (30.7)	6788 (28.7)	5157 (29.8)	4320 (30.4)	4111 (29.3)	3873 (27.8)
Total deaths	6803 (16.1)	333 (28.4)	4226 (17.9)	2244 (12.9)	2018 (14.4)	2381 (17.0)	2404 (17.1)
T2D deaths	248 (0.6)	13 (1.1)	163 (0.7)	72 (0.4)	82 (0.6)	85 (0.6)	81 (0.6)

^a^ Values are numbers (percentages) unless stated otherwise. ^b^ Tertile 1 of E-DII ranged from −5.44 to −0.50 in UK Biobank, and from −5.28 to 0.83 in US NHANES; tertile 2 of E-DII ranged from −0.50 to 1.13 in UK Biobank and from 0.83 to 2.54 in US NHANES; tertile 3 of E-DII ranged from 1.13 to 4.65 in UK Biobank and from 2.54 to 5.79 in US NHANES. Abbreviations: DDS, dietary diversity score; E-DII, energy-adjusted dietary inflammatory index; SD, standard deviation; PA, physical activity; MET, metabolic equivalent; BMI, body mass index; CVD, cardiovascular disease.

**Table 2:** Associations of DDS with outcomes in the UK Biobank and US NHANES.

	Per 1-Point Increase in DDS (HRs, 95% CIs)	DDS Categories (HRs, 95% CIs)			*p*_trend_
		Low DDS ^b^ (1–6)	Medium DDS ^b^ (7–12)	High DDS ^b^ (13–18)	
**All-cause Mortality**					
UK Biobank					
Events (*n*)	8338	1218	5201	1919	
Model 1 ^a^	0.957 (0.950, 0.964)	1.00	0.79 (0.74, 0.84)	0.66 (0.62, 0.71)	<0.001
Model 2 ^a^	0.961 (0.954, 0.968)	1.00	0.81 (0.76, 0.86)	0.69 (0.64, 0.74)	<0.001
US NHANES					
Events (*n*)	6803	333	4226	2244	
Model 1 ^a^	0.952 (0.942, 0.962)	1.00	0.82 (0.73, 0.91)	0.71 (0.63, 0.80)	<0.001
Model 2 ^a^	0.956 (0.946, 0.966)	1.00	0.78 (0.70, 0.88)	0.69 (0.61, 0.78)	<0.001
**T2D mortality**					
UK Biobank					
Events (*n*)	353	54	208	91	
Model 1 ^a^	0.984 (0.950, 1.020)	1.00	0.79 (0.58, 1.08)	0.85 (0.60, 1.21)	0.496
Model 2 ^a^	1.001 (0.966, 1.037)	1.00	0.87 (0.64, 1.19)	0.99 (0.70, 1.41)	0.930
US NHANES					
Events (*n*)	248	13	163	72	
Model 1 ^a^	0.954 (0.906, 1.004)	1.00	0.94 (0.53, 1.67)	0.76 (0.41, 1.41)	0.099
Model 2 ^a^	0.967 (0.915, 1.023)	1.00	0.87 (0.49, 1.56)	0.72 (0.38, 1.34)	0.092
**Incident T2D in UK Biobank**					
Events (*n*)	3416	577	2077	762	
Model 1 ^a^	0.955 (0.944, 0.966)	1.00	0.75 (0.68, 0.83)	0.66 (0.59, 0.74)	<0.001
Model 2 ^a^	0.974 (0.963, 0.985)	1.00	0.84 (0.76, 0.92)	0.79 (0.70, 0.88)	<0.001

^a^ HRs (95% CIs) of DDS with outcomes were examined using Cox proportional hazards regression models; model 1 was adjusted for age, sex, race, household income, residence (UK Biobank only), family history of diabetes (included in analyses when using incidence or mortality of T2D as outcomes), smoking status, alcohol consumption, physical activity, BMI, and total calorie intake from diet; model 2 additionally included dietary supplements, CVD, cancer, hypertension, and hyperlipidemia. ^b^ DDS categories (low (1–6), medium (7–12), and high (13–18)) were defined according to practical implications for public health. Abbreviations: CIs, confidence intervals; DDS, dietary diversity score; HRs, hazard ratios; T2D, type 2 diabetes.

**Table 3:** Associations of E-DII with outcomes in the UK Biobank and US NHANES.

	Per 1-Point Decrease in E-DII (HRs, 95% CIs)	E-DII (HRs, 95% CIs)			*p*_trend_
		Tertile 3	Tertile 2	Tertile 1	
**All-cause Mortality**					
UK Biobank					
Events (*n*)	8338	2814	2658	2866	
Model 1 ^a^	0.958 (0.944, 0.973)	1.00	0.87 (0.82, 0.92)	0.84 (0.79, 0.90)	<0.001
Model 2 ^a^	0.962 (0.948, 0.976)	1.00	0.88 (0.83, 0.93)	0.86 (0.81, 0.91)	<0.001
US NHANES					
Events (*n*)	6803	2018	2381	2404	
Model 1 ^a^	0.944 (0.929, 0.959)	1.00	0.97 (0.91, 1.02)	0.81 (0.76, 0.87)	<0.001
Model 2 ^a^	0.950 (0.935, 0.965)	1.00	0.98 (0.92, 1.04)	0.83 (0.77, 0.88)	<0.001
**T2D mortality**					
UK Biobank					
Events (*n*)	353	122	95	136	
Model 1 ^a^	0.950 (0.883, 1.021)	1.00	0.87 (0.65, 1.16)	0.72 (0.51, 1.01)	0.036
Model 2 ^a^	0.966 (0.899, 1.038)	1.00	0.93 (0.70, 1.24)	0.75 (0.53, 1.02)	0.045
US NHANES					
Events (*n*)	248	82	85	81	
Model 1 ^a^	1.059 (0.975, 1.149)	1.00	1.29 (0.91, 1.83)	1.15 (0.84, 1.57)	0.236
Model 2 ^a^	1.084 (0.999, 1.177)	1.00	1.39 (0.98, 1.98)	1.18 (0.86, 1.62)	0.112
**Incident T2D in UK Biobank**					
Events (*n*)	3416	1159	1033	1224	
Model 1 ^a^	0.948 (0.926, 0.970)	1.00	0.83 (0.76, 0.92)	0.81 (0.75, 0.89)	<0.001
Model 2 ^a^	0.967 (0.945, 0.989)	1.00	0.90 (0.82, 0.97)	0.86 (0.79, 0.94)	0.032

^a^ HRs (95% CIs) of E-DII with outcomes were examined using Cox proportional hazards regression models; model 1 was adjusted for age, sex, race, household income, residence (UK Biobank only), family history of diabetes (included in analyses when using incidence or mortality of T2D as outcomes), smoking status, alcohol consumption, physical activity, BMI, and total calorie intake from diet; model 2 additionally included dietary supplements, CVD, cancer, hypertension, and hyperlipidemia. Abbreviations: CIs, confidence intervals; E-DII, energy-adjusted dietary inflammatory index; HRs, hazard ratios; T2D, type 2 diabetes.

**Table 4:** Combined effects of DDS and E-DII with outcomes in the UK Biobank and US NHANES.

DDS Categories ^a^	E-DII (HRs, 95% CIs) ^a,b^			RERI ^c^		*p*_interaction_^d^
	Tertile 3	Tertile 2	Tertile 1	Tertile 2 of E-DII	Tertile 1 of E-DII	
**All-cause mortality**						
UK Biobank						0.119
Low DDS	1.00	0.87 (0.75, 1.00)	0.90 (0.72, 1.11)			
Medium DDS	0.80 (0.74, 0.87)	0.77 (0.71, 0.84)	0.75 (0.69, 0.82)	0.11 (−0.03, 0.24)	−0.06 (−0.15, 0.26)	
High DDS	0.72 (0.63, 0.83)	0.66 (0.60, 0.73)	0.61 (0.55, 0.68)	0.03 (−0.13, 0.18)	0.04 (−0.18, 0.26)	
US NHANES						0.950
Low DDS	1.00	0.99 (0.72, 1.36)	0.80 (0.61, 1.05)			
Medium DDS	0.78 (0.67, 0.90)	0.77 (0.66, 0.89)	0.66 (0.57, 0.77)	0.16 (−0.06, 0.39)	−0.01 (−0.30, 0.27)	
High DDS	0.71 (0.61, 0.83)	0.68 (0.58, 0.80)	0.58 (0.49, 0.68)	0.21 (−0.01, 0.44)	0.03 (−0.26, 0.31)	
**T2D mortality**						
UK Biobank						0.399
Low DDS	1.00	1.05 (0.54, 2.03)	1.07 (0.38, 3.07)			
Medium DDS	1.03 (0.70, 1.52)	0.72 (0.47, 1.09)	0.90 (0.58, 1.39)	−0.37 (−1.16, 0.43)	−0.21 (−1.37, 0.96)	
High DDS	1.17 (0.65, 2.10)	0.81 (0.48, 1.36)	1.07 (0.68, 1.69)	−0.40 (−1.40, 0.59)	−0.16 (−1.45, 1.12)	
US NHANES						0.092
Low DDS	1.00	1.13 (0.66, 1.99)	1.79 (0.40, 3.93)			
Medium DDS	1.00 (0.44, 2.39)	1.12 (0.46, 2.63)	1.63 (0.65, 3.24)	−1.02 (−0.49, 0.46)	−0.23 (−0.93, 0.47)	
High DDS	0.93 (0.37, 2.30)	1.15 (0.45, 2.87)	0.93 (0.37, 2.31)	−0.84 (−2.27, 1.17)	−0.76 (−2.65, 0.83)	
**Incident T2D in UK Biobank**						0.097
Low DDS	1.00	0.96 (0.78, 1.17)	1.11 (0.82, 1.50)			
Medium DDS	0.90 (0.80, 1.02)	0.83 (0.72, 0.95)	0.78 (0.69, 0.89)	−0.08 (−0.29, 0.14)	−0.18 (−0.52, 0.16)	
High DDS	0.80 (0.69, 0.93)	0.79 (0.64, 0.94)	0.75 (0.64, 0.89)	−0.00 (−0.26, 0.26)	−0.13 (−0.59, 0.33)	

^a^ DDS categories (low (1–6), medium (7–12), and high (13–18)) were defined according to practical implications for public health; tertile 1 of E-DII ranged from −5.44 to −0.50 in UK Biobank and from −5.28 to 0.83 in US NHANES; tertile 2 of E-DII ranged from −0.50 to 1.13 in UK Biobank and from 0.83 to 2.54 in US NHANES; tertile 3 of E-DII ranged from 1.13 to 4.65 in UK Biobank and from 2.54 to 5.79 in US NHANES. ^b^ All results were calculated based on covariates in model 2: age, sex, race, household income, residence (UK Biobank only), family history of diabetes (included in analyses when using incidence or mortality of T2D as outcomes), smoking status, alcohol consumption, physical activity, BMI, total calorie intake from diet, dietary supplement, CVD, cancer, hypertension, and hyperlipidemia. ^c^ The estimates of RERI were calculated based on the reference group with low DDS and tertile 3 of E-DII. ^d^ Likelihood tests were applied to test the significance of the interaction term by comparing the model with and without the interaction term. Abbreviations: CIs, confidence intervals; DDS, dietary diversity score; E-DII, energy-adjusted dietary inflammatory index; HRs, hazard ratios; T2D, type 2 diabetes.

## Data Availability

The datasets generated and analyzed during the current study are available upon reasonable request to the Access Management System (AMS) through the UK Biobank website (https://www.ukbiobank.ac.uk/enable-your-research/apply-for-access).

## Supplementary Materials

The following supporting information can be downloaded at: https://www.mdpi.com/article/10.3390/nu15092120/s1, Table S1: Food items and mixed dishes involved in calculating DDS using 24-hour dietary recall information in UK Biobank and US NHANES; Table S2: Dietary components for E-DII calculation in UK Biobank and US NHANES; Table S3: Sensitivity analysis of HRs (95% CIs) of DDS with outcomes after excluding events occurred in the first two years of follow-up in UK Biobank and US NHANES; Table S4: Sensitivity analysis of HRs (95% CIs) of E-DII with outcomes after excluding events occurred in the first two years of follow-up in UK Biobank and US NHANES; Table S5: Sensitivity analysis of combined associations of DDS and E-DII with outcomes after excluding events occurred in the first two years of follow-up in UK Biobank and US NHANES; Table S6: Sensitivity analysis of HRs (95% CIs) of DDS and E-DII and outcomes after excluding participants between the ages of 20 and 40 in US NHANES; Table S7: Sensitivity analysis of combined associations of DDS and E-DII with outcomes after excluding participants between the ages of 20 and 40 in US NHANES; Table S8: Sensitivity analysis of HRs (95% CIs) of DDS with outcomes when using the FFQ information to calculate DDS in UK Biobank; Table S9: Subgroup analyses of HRs (95% CIs) of DDS and E-DII with outcomes in UK Biobank; Table S10: Subgroup analysis of HRs (95% CIs) of DDS and E-DII with outcomes in US NHANES; Figure S1: The time points of the five rounds of dietary surveys in UK Biobank; Figure S2: Flow chart for UK Biobank; Figure S3: Flow chart for US NHANES; Figure S4: Restricted cubic spline plot of association of DDS with all-cause mortality and incidence and mortality of T2D in UK Biobank and US NHANES; Figure S5: Restricted cubic spline plot of association of E-DII with all-cause mortality and incidence and mortality of T2D in UK Biobank and US NHANES.

## Abbreviations

BMI; body mass index; CI; confidence interval; CVD; cardiovascular diseases; DDS; diversity score; DALYs, disability-adjusted life years; DII; dietary inflammatory index; E-DII; energy-adjusted dietary inflammatory index; HR; hazard ratio; Ref; reference group; T2D; type 2 diabetes.
