# Supplementary material for: Dietary Diversity and Inflammatory Diet Associated with All-Cause Mortality and Incidence and Mortality of Type 2 Diabetes: Two Prospective Cohort Studies
Source: Nutrients. 2023 Apr 28;15(9):2120. doi: 10.3390/nu15092120 (PMC10180882; doi:10.3390/nu15092120)
Supplement: Supplementary file 1 [file nutrients-15-02120-s001.zip › nutrients-2267846-supplementary.pdf]

Supplemental Material

Dietary diversity and inflammatory diet associated with all-cause mortality and incidence and mortality of type 2 diabetes: two prospective cohort studies

Guzhengyue Zheng, Miao Cai, Huiling Liu, Rui Li, Zhengmin (Min) Qian, Steven W. Howard, Amy E. Keith, Shiyu Zhang, Xiaojie Wang, Junguo Zhang, Hualiang Lin, Junjie Hua *

Supplemental methods Assessment of dietary diversity score (DDS) based on food frequency questionnaires (FFQ) in UK Biobank

In UK Biobank, 502,461 participants completed a brief touchscreen food frequency questionnaire (FFQ) with 33 dietary information about beverages, main foods, and food consumed over the previous year at baseline. Among the 502,461 adults, participants meeting the following criteria were excluded in this study: whose dietary information was missing or unbelievable, who had withdrawn consent for linkage, who had missing data on key covariates, or who with occurrences of all types of diabetes (type 1 diabetes, type 2 diabetes, gestational diabetes, other types of diabetes) at baseline. The remaining 431,647 participants were included in the analysis.

DDS was assessed based on FFQ, related to the 15 major food groups: processed meat, beef, mutton, pork, poultry, oily fish, non-oily fish, cheese, milk, cereal, bread, cooked

vegetables, raw vegetables, fresh fruits, and dried fruits. Participants were asked how often processed meat, beef, mutton, pork, poultry, oily fish, non-oily fish, and cheese were consumed with eight choices: “never eaten”, “<1 time a week”, “1 time a week”, “2-4 times a week”, “5-6 times a week”, “≥1 time daily”, “do not know”, and “prefer not to answer”. For cereal, bread, cooked vegetables, raw vegetables, fresh fruit, and dried fruit, participants answered the integer number of bowls/slices/heaped tablespoons/pieces of each item. The participants also had three other options to select: “less than one”, “do not know”, or “prefer not to answer”. Responses of “do not know” or “prefer not to answer” for a specific dietary item were converted into missing values. Consumption of any food group without considering a minimum amount was categorized as one point. We consider that a person's normal diet is impossible to eat nothing for a day, the DDS ranges from 1-15. A higher DDS score reflects a richer diet and vice versa. We divided DDS score into three categories: low DDS (ranges 1-5), medium DDS (ranges 6-10), and high DDS (ranges 11-15) and the reference group was set as the participants with low DDS category.

Table S1 Food items and mixed dishes involved in calculating DDS using 24-hour dietary recall information in UK Biobank and US NHANES

**Major food groups**	**Subgroups**	**Representative food items and mixed dishes on the 24-hour dietary recalls**
**Grain products**	Whole grains	Porridge, oat crunch, bran cereal, whole-wheat cereal, whole meal sliced bread, whole meal baguette, whole meal bap, whole meal bread roll, crispbread, oat cakes, whole meal pasta, brown rice, couscous, other whole grains
	Non-whole grains	Muesli, plain cereal, dried fruit cereal, plain sliced bread, plain baguette, plain bap, plain bread roll, naan bread, garlic bread, other non-whole grain bread, white pasta, white rice, sushi, snack pot, pizza, pancakes, scotch pancakes, Danish pastry, cake, other non-whole grains
**Vegetables**	Dark green leafy	Spinach, broccoli, watercress, beetroot
	Vitamin A-rich	Carrots, sweet peppers
	Starchy tubers	Potatoes, boiled potatoes, mashed potatoes, sweet potatoes, butternut squash, sweet corn
	Other	Vegetable pieces, (mixed) salad, cabbage, cauliflower, celery, courgetti, cucumber, garlic,

		leeks, lettuce, mushrooms, onions, parsnip, fresh tomatoes, tinned tomatoes, turnip, other vegetables
**Fruits**	Citrus	Grape, oranges, satsuma
	Vitamin A-rich	Melon, peach
	Other	Mixed fruits, bananas, berry, cherry, grape, mango, pear, pineapple, plum, olives, avocados, other fruits
**Meat and protein alternatives**	Red meat	Beef, pork, mutton, burger, bacon, ham, corned beef, sausages, lasagna, meat soup, quiche, savory pies
	Poultry	Deep fried chicken, chicken, other poultry
	Fish and seafood	Tinned tuna, oily fish, breaded fish, battered fish, white fish, prawns, lobster, shellfish, other fish
	Organ meat	Liver, kidney
	Eggs	Whole eggs, scrambled eggs, eggs in sandwiches, scotch eggs, other eggs
	Legumes and nuts	Soya dessert, salted peanuts, unsalted peanuts, salted nuts, unsalted nuts, seeds, tofu, baked beans, broad beans, green beans, other beans, peas

**Dairy products**	Milk	Milk (fortified, goat's, sheep's, etc.), added milk to coffee, added milk to tea, ice cream, lasagna, quiche
	Yoghurt	Yogurt smoothie, flavored milk, ice cream, yogurt (whole milk, low fat)
	Cheese	Cheese, low fat (spreadable) cheese, hard cheese, soft cheese, blue cheese, spreadable cheese, cottage cheese, feta, mozzarella, goat cheese, other cheese

Abbreviation: DDS, dietary diversity score.

Table S2 Dietary components for E-DII calculation in UK Biobank and US NHANES

	Dietary components
**UK Biobank**	Energy, alcohol, total fat, protein, beta-carotene, carbohydrate, cholesterol, fiber, folate, iron, magnesium, MUFA, PUFA, niacin, riboflavin, saturated fat, selenium, thiamin, Vitamin A, Vitamin B_{12}, Vitamin B_6, Vitamin C, Vitamin D, Vitamin E, zinc, n-3 fatty acids, n-6 fatty acids, trans fat[a]
**US NHANES**	Energy, alcohol, total fat, protein, beta-carotene, carbohydrate, cholesterol, fiber, folate, iron, magnesium, MUFA, PUFA, niacin, riboflavin, saturated fat, selenium, thiamin, Vitamin A, Vitamin B_{12}, Vitamin B_6, Vitamin C, Vitamin D, Vitamin E, zinc, n-3 fatty acids, n-6 fatty acids, caffeine [a]

[a] One of the 28 dietary components were different: trans fats in UK Biobank and caffeine in US NHANES.

Abbreviation: E-DII, energy-adjusted dietary inflammatory index; MUFA, monounsaturated fatty acids; PUFA, polyunsaturated fatty acids.

Table S3 Sensitivity analysis of HRs (95% CIs) of DDS with outcomes after excluding events occurred in the first two years of follow-up in UK Biobank and US NHANES

	DDS (HRs, 95% CIs)			
	**Per 1-point increase in DDS**	**Low (1-6)**	**Medium (7-12)**	**High (13-18)**
**All-cause mortality**				
UK Biobank				
Model 1 [a]	0.963 (0.956, 0.970)	1.00	0.81 (0.76, 0.86)	0.70 (0.65, 0.75)
Model 2 [a]	0.966 (0.959, 0.973)	1.00	0.82 (0.77, 0.88)	0.72 (0.66, 0.77)
US NHANES				
Model 1 [a]	0.958 (0.947, 0.969)	1.00	0.81 (0.72, 0.92)	0.70 (0.62, 0.80)
Model 2 [a]	0.756 (0.945, 0.967)	1.00	0.78 (0.69, 0.88)	0.68 (0.60, 0.78)
**T2D mortality**				
UK Biobank				
Model 1 [a]	0.985 (0.949, 1.022)	1.00	0.74 (0.54,1.02)	0.84 (0.59, 1.21)
Model 2 [a]	1.001 (0.965, 1.039)	1.00	0.81 (0.59, 1.11)	0.98 (0.68, 1.40)
US NHANES				
Model 1[a]	0.974 (0.920, 1.030)	1.00	0.86 (0.48, 1.52)	0.76 (0.40, 1.41)
Model 2[a]	0.959 (0.904, 1.016)	1.00	0.68 (0.38, 1.22)	0.60 (0.32, 1.14)
**Incident T2D in UK Biobank**				
Model 1 [a]	0.966 (0.955, 0.978)	1.00	0.81 (0.73, 0.89)	0.74 (0.66, 0.83)

Model 2 [a]	0.977 (0.965, 0.988)	1.00	0.85 (0.77, 0.94)	0.81 (0.72, 0.91)

[a] HRs (95% CIs) of DDS with outcomes were examined using Cox proportional hazards regression models; model 1 was adjusted for age, sex, race, household income, residence (UK Biobank only), family history of diabetes (included in analyses when using incidence or mortality of T2D as outcomes), smoking status, alcohol consumption, physical activity, BMI, and total calorie intake from diet; model 2 additionally included dietary supplement, CVD, cancer, hypertension, and hyperlipidemia. Abbreviations: CIs, confidence intervals; DDS, dietary diversity score; HRs, hazard ratios; T2D, type 2 diabetes.

Table S4 Sensitivity analysis of HRs (95% CIs) of E-DII with outcomes after excluding events occurred in the first two years of follow-up in UK Biobank and US NHANES

	E-DII (HRs, 95% CIs)			P_{trend}
	**Tertile 3**	**Tertile 2**	**Tertile 1**	
**All-cause mortality**				
UK Biobank				
Model 1 [a]	1.00	0.89 (0.84, 0.94)	0.87 (0.83, 0.92)	<0.001
Model 2 [a]	1.00	0.89 (0.84, 0.94)	0.86 (0.81, 0.92)	<0.001
US NHANES				
Model 1 [a]	1.00	0.95 (0.89, 1.01)	0.81 (0.76, 0.88)	<0.001
Model 2 [a]	1.00	0.95 (0.90, 1.02)	0.82 (0.77, 0.89)	<0.001
**T2D mortality**				
UK Biobank				
Model 1 [a]	1.00	0.83 (0.62, 1.13)	0.69 (0.52, 0.92)	0.262
Model 2 [a]	1.00	0.90 (0.66, 1.22)	0.74 (0.55, 0.98)	0.522
US NHANES				
Model 1 [a]	1.00	1.20(0.86, 1.66)	1.33 (0.93, 1.94)	0.237
Model 2 [a]	1.00	1.16 (0.84, 1.62)	1.30 (0.90, 1.87)	0.085
**Incident T2D in UK Biobank**				
Model 1 [a]	1.00	0.83 (0.75, 0.91)	0.81 (0.74, 0.89)	<0.001
Model 2 [a]	1.00	0.89 (0.81, 0.98)	0.86 (0.79, 0.94)	0.028

[a] HRs (95% CIs) of E-DII with outcomes were examined using Cox proportional hazards regression models; model 1 was adjusted for age, sex, race, household income, residence (UK Biobank only), family history of diabetes (included in analyses when using incidence or mortality of T2D as outcomes), smoking status, alcohol consumption, physical activity, BMI, and total calorie intake from diet; model 2 additionally included dietary supplement, CVD, cancer, hypertension, and hyperlipidemia.

Abbreviations: CIs, confidence intervals; E-DII, energy-adjusted dietary inflammatory index; HRs, hazard ratios; T2D, type 2 diabetes.

Table S5 Sensitivity analysis of combined associations of DDS and E-DII with outcomes after excluding events occurred in the first two years of follow-up in UK Biobank and US NHANES

**DDS categories** [a]	**E-DII (HRs, 95% CIs)** [a, b]		
	**Tertile 3**	**Tertile 2**	**Tertile 1**
**All-cause mortality**			
UK Biobank			
Low DDS	1.00	0.85 (0.73, 0.98)	0.92 (0.72, 1.14)
Medium DDS	0.81 (0.74, 0.87)	0.79 (0.71, 0.85)	0.75 (0.69, 0.83)
High DDS	0.76 (0.66, 0.85)	0.65 (0.58, 0.72)	0.68 (0.61, 0.75)
US NHANES			
Low DDS	1.00	0.77 (0.59, 1.01)	0.94 (0.66, 1.30)
Medium DDS	0.78 (0.67, 0.90)	0.75 (0.64, 0.87)	0.66 (0.56, 0.79)
High DDS	0.70 (0.58, 0.83)	0.69 (0.58, 0.80)	0.59 (0.50, 0.70)
**T2D mortality**			
UK Biobank			
Low DDS	1.00	0.99 (0.67, 1.46)	0.97 (0.55, 1.71)
Medium DDS	1.07 (0.85, 1.35)	0.99 (0.78, 1.25)	0.94 (0.72, 1.20)
High DDS	1.41 (0.99, 2.00)	1.07 (0.80, 1.45)	1.01 (0.77, 1.32)
US NHANES			
Low DDS	1.00	1.34 (0.67, 2.69)	1.51 (0.61, 3.73)
Medium DDS	0.86 (0.55, 1.38)	1.03 (0.65, 1.65)	1.09 (0.68, 1.77)
High DDS	0.84 (0.50, 1.41)	0.98 (0.59, 1.61)	0.87 (0.53, 1.46)

**Incident T2D in UK Biobank**			
Low DDS	1.00	0.98 (0.79, 1.20)	1.13 (0.84, 1.55)
Medium DDS	0.91 (0.80, 1.03)	0.80 (0.70, 0.90)	0.85 (0.73, 0.97)
High DDS	0.83 (0.65, 1.02)	0.77 (0.66, 0.93)	0.82 (0.70, 0.96)

[a] DDS categories (low (1−6), medium (7-12), and high (13-18)) were defined according to practical implications for public health; Tertile 1 of E-DII ranged from -5.44 to -0.50 in UK Biobank, and from -5.28 to 0.83 in US NHANES; tertile 2 of E-DII ranged from -0.50 to 1.13 in UK Biobank, and from 0.83 to 2.54 in US NHANES; tertile 3 of E-DII ranged from 1.13 to 4.65 in UK Biobank, and from 2.54 to 5.79 in US NHANES.

[b] All results were calculated based on covariates in model 2: age, sex, race, household income, residence (UK Biobank only), family history of diabetes (included in analyses when using incidence or mortality of T2D as outcomes), smoking status, alcohol consumption, physical activity, BMI, total calorie intake from diet, dietary supplement, CVD, cancer, hypertension, and hyperlipidemia.

Abbreviations: CIs, confidence intervals; DDS, dietary diversity score; E-DII, energy-adjusted dietary inflammatory index; HRs, hazard ratios; T2D, type 2 diabetes.

Table S6 Sensitivity analysis of HRs (95% CIs) of DDS and E-DII and outcomes after excluding participants between the ages of 20 and 40 in US NHANES

	All-cause mortality (HRs, 95% CIs)		T2D mortality (HRs, 95% CIs)	
	**Model 1 [c]**	**Model 2 [c]**	**Model 1 [c]**	**Model 2 [c]**
DDS [a]				
Low [a]	1.00	1.00	1.00	1.00
Medium [a]	0.80 (0.71, 0.90)	0.79 (0.70, 0.89)	0.89 (0.49, 1.60)	0.75 (0.41, 1.38)
High [a]	0.68 (0.60, 0.77)	0.70 (0.61, 0.79)	0.66 (0.36, 1.25)	0.60 (0.31, 1.15)
E-DII [b]				
Tertile 3 [b]	1.00	1.00	1.00	1.00
Tertile 2 [b]	0.97 (0.92, 1.03)	0.98 (0.92, 1.04)	1.16 (0.84, 1.59)	1.13 (0.82, 1.55)
Tertile 1 [b]	0.81 (0.76, 0.87)	0.82 (0.77, 0.88)	1.27 (0.89, 1.82)	1.24 (0.87, 1.78)

[a] DDS categories (low (1-6), medium (7-12), and high (13-18)) were defined according to practical implications for public health.

[b] Tertile 1 of E-DII ranged from -5.28 to 0.83, tertile 2 of E-DII ranged from 0.83 to 2.54, and tertile 3 of E-DII ranged from 2.54 to 5.79 in US NHANES.

[c] HRs (95% CIs) of DDS and E-DII with outcomes were examined using Cox proportional hazards regression models; model 1 was adjusted for age, sex, race, household income, , family history of diabetes (included in analyses when using mortality of T2D as outcomes), smoking status, alcohol consumption, physical activity, BMI, and total calorie intake from diet; model 2 additionally included dietary supplement, CVD, cancer, hypertension, and hyperlipidemia.

Abbreviations: CI, confidence interval; DDS, dietary diversity score; E-DII, dietary inflammatory index;

HRs, hazard ratios; T2D, type 2 diabetes.

Table S7 Sensitivity analysis of combined associations of DDS and E-DII with outcomes after excluding participants between the ages of 20 and 40 in US NHANES

	All-cause mortality (HRs, 95% CIs) [c]			T2D mortality (HRs, 95% CIs) [c]		
	**Low DDS [a]**	**Medium DDS [a]**	**High DDS [a]**	**Low DDS [a]**	**Medium DDS [a]**	**High DDS [a]**
E-DII [b]						
Tertile 3 [b]	1.00	0.83 (0.63, 1.10)	0.99 (0.70, 1.37)	1.00	1.40 (0.68, 2.88)	1.58 (0.63, 3.98)
Tertile 2 [b]	0.79 (0.68, 0.93)	0.77 (0.67, 0.90)	0.69 (0.58, 0.80)	0.96 (0.60, 1.52)	1.19 (0.74, 1.90)	1.18 (0.70, 1.90)
Tertile 1 [b]	0.74 (0.62, 0.87)	0.70 (0.59, 0.83)	0.60 (0.50, 0.70)	0.87 (0.51, 1.47)	1.02 (0.60, 1.69)	0.86 (0.51, 1.44)

[a] DDS categories (low (1-6), medium (7-12), and high (13-18)) were defined according to practical implications for public health.

[b] Tertile 1 of E-DII ranged from -5.28 to 0.83, tertile 2 of E-DII ranged from 0.83 to 2.54, and tertile 3 of E-DII ranged from 2.54 to 5.79 in US NHANES.

[c] All results were calculated based on covariates in model 2: age, sex, race, household income, family history of diabetes (included in analyses when using mortality of T2D as outcomes), smoking status, alcohol consumption, physical activity, BMI, total calorie intake from diet, dietary

supplement, CVD, cancer, hypertension, and hyperlipidemia.

Abbreviations: CIs, confidence intervals; DDS, dietary diversity score; E-DII, energy-adjusted dietary inflammatory index; HRs, hazard ratios; T2D, type 2 diabetes.

Table S8 Sensitivity analysis of HRs (95% CIs) of DDS with outcomes when using the FFQ information to calculate DDS in UK Biobank

	DDS (HRs, 95% CIs)		
	**Low (1-5)**	**Medium (6-10)**	**High (11-15)**
All-cause mortality			
Model 1 [a]	1.00	0.90 (0.87,0.94)	0.92 (0.86, 0.98)
Model 2 [a]	1.00	0.90 (0.87, 0.93)	0.90 (0.85, 0.96)
Incident T2D			
Model 1 [a]	1.00	0.94 (0.89, 1.00)	1.05 (0.95, 1.16)
Model 2 [a]	1.00	0.89 (0.84, 0.94)	0.91 (0.83, 1.01)
T2D mortality			
Model 1 [a]	1.00	0.94 (0.86, 1.02)	1.00 (0.88, 1.14)
Model 2 [a]	1.00	0.92 (0.84, 1.01)	1.00 (0.87, 1.13)

[a] HRs (95% CIs) of DDS with outcomes were examined using Cox proportional hazards regression models; model 1 was adjusted for age, sex, race, household income, residence, family history of diabetes (included in analyses when using incidence and mortality of T2D as outcomes), smoking status, alcohol consumption, physical activity, BMI, and total calorie intake from diet; model 2 additionally included dietary supplement, CVD, cancer, hypertension, and hyperlipidemia.

Abbreviations: CIs, confidence intervals; DDS, dietary diversity score; FFQ, food frequency questionnaire; HRs, hazard ratios; T2D, type 2 diabetes.

Table S9 Subgroup analyses of HRs (95% CIs) of DDS and E-DII with outcomes in UK Biobank [a]

	All-cause mortality (HRs, 95% CIs)		Incident T2D (HRs, 95% CIs)		T2D mortality (HRs, 95% CIs)	
	**Per 1-point increase in DDS**	**Per 1-point decrease in E-DII**	**Per 1-point increase in DDS**	**Per 1-point decrease in E-DII**	**Per 1-point increase in DDS**	**Per 1-point decrease in E-DII**
Age						
<55	0.98 (0.97, 1.00)	0.98 (0.94, 1.02)	0.97 (0.95, 0.99)	0.94 (0.90, 0.98)	0.99 (0.91, 1.08)	0.88 (0.74, 1.04)
≥55	0.96 (0.95, 0.97)	0.96 (0.94, 0.98)	0.97 (0.96,0.98)	0.98 (0.95, 1.01)	1.00 (0.96, 1.04)	0.99 (0.92, 1.07)
$P_{interaction}$	0.019	0.828	0.169	0.104	0.954	0.633
Sex						
Male	0.95 (0.94, 0.96)	0.95 (0.93, 0.97)	0.98 (0.96, 0.99)	0.98 (0.95, 1.01)	0.99 (0.95, 1.03)	0.94 (0.86, 1.03)
Female	0.97 (0.96, 0.98)	0.97 (0.95, 1.00)	0.96 (0.94, 0.98)	0.94 (0.91, 0.97)	1.00 (0.95, 1.07)	1.00 (0.89, 1.13)
$P_{interaction}$	0.017	0.128	0.481	0.482	0.668	0.368
BMI						

< 25 kg/m^2	0.94 (0.93, 0.96)	0.96 (0.93, 0.99)	0.94 (0.91, 0.97)	0.94 (0.88, 1.01)	0.90 (0.82, 1.00)	0.82 (0.66, 1.01)
≥25 kg/m^2	0.96 (0.95, 0.97)	0.96 (0.94, 0.98)	0.96 (0.95, 0.97)	0.96 (0.94, 0.99)	1.00 (0.96, 1.03)	0.98 (0.91, 1.06)
$P_{interaction}$	<0.001	0.125	0.077	0.071	0.370	0.219

[a] All results were calculated based on covariates in model 2: age, sex, race, household income, residence, family history of diabetes (included in analyses when using incidence or mortality of T2D as outcomes), smoking status, alcohol consumption, physical activity, BMI, total calorie intake from diet, dietary supplement, CVD, cancer, hypertension, and hyperlipidemia.

Abbreviations: CIs, confidence intervals; DDS, dietary diversity score; E-DII, energy-adjusted dietary inflammatory index; HRs, hazard ratios; T2D, type 2 diabetes.

Table S10 Subgroup analysis of HRs (95% CIs) of DDS and E-DII with outcomes in US NHANES [a]

	All-cause mortality (HRs, 95% CIs)		T2D mortality (HRs, 95% CIs)	
	**Per 1-point increase in DDS**	**Per 1-point decrease in E-DII**	**Per 1-point increase in DDS**	**Per 1-point decrease in E-DII**
Age				
<55	0.95 (0.92, 0.97)	0.99 (0.94, 1.03)	1.01 (0.88, 1.17)	1.21 (0.98, 1.49)
≥55	0.98 (0.97, 0.99)	0.97 (0.95, 0.99)	0.95 (0.89, 1.01)	1.06 (0.96, 1.16)
$P_{interaction}$	< 0.001	0.052	0.100	0.573
Sex				
Male	0.96 (0.94, 0.97)	0.94 (0.92, 0.96)	0.96 (0.89, 1.04)	1.04 (0.93, 1.17)
Female	0.96 (0.94, 0.97)	0.97 (0.95, 0.99)	0.95 (0.88, 1.03)	1.11 (0.98, 1.26)
$P_{interaction}$	0.842	0.020	0.985	0.538
BMI				
<25 kg/m^2	0.95 (0.94, 0.97)	0.93 (0.90, 0.95)	0.87 (0.77, 0.98)	0.89 (0.74, 1.07)
≥25 kg/m^2	0.96 (0.94, 0.97)	0.96 (0.94, 0.97)	0.98 (0.92, 1.04)	1.12 (1.02, 1.22)
$P_{interaction}$	0.205	0.208	0.816	0.182

[a] All results were calculated based on covariates in model 2: age, sex, race, household income, , family history of diabetes (included in analyses when using mortality of T2D as outcomes), smoking status, alcohol consumption, physical activity, BMI, total calorie intake from diet, dietary supplement, CVD, cancer, hypertension, and hyperlipidemia.

Abbreviations: CIs, confidence intervals; DDS, dietary diversity score; E-DII, energy-adjusted dietary inflammatory index; HRs, hazard ratios; T2D, type 2 diabetes.

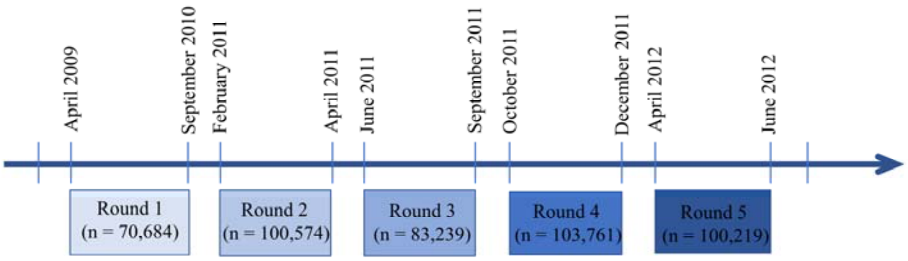

Figure S1 The time points of the five rounds of dietary surveys in UK Biobank

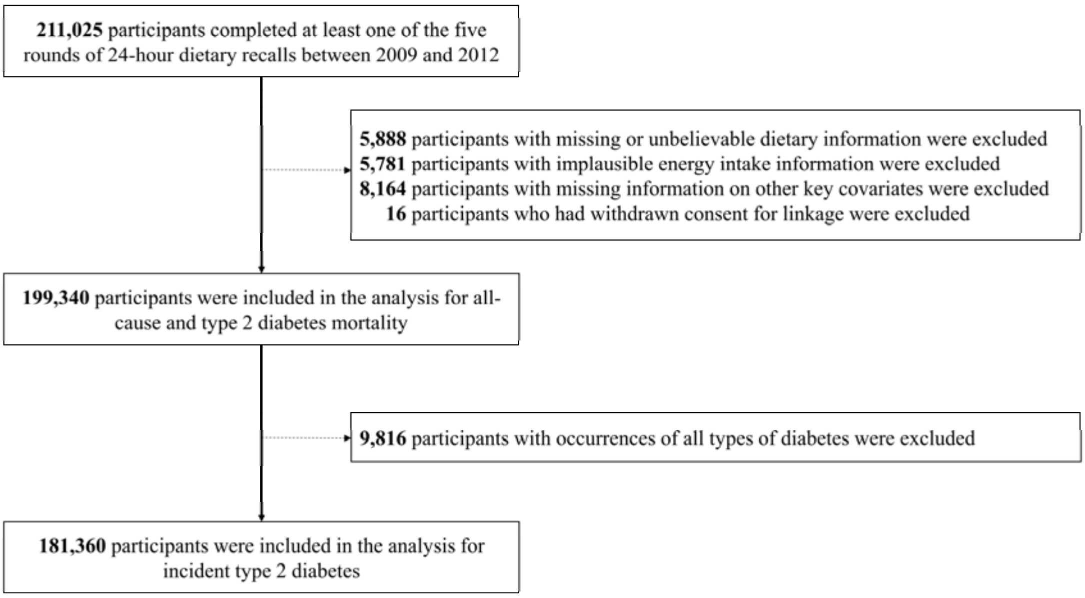

Figure S2 Flow chart for UK Biobank

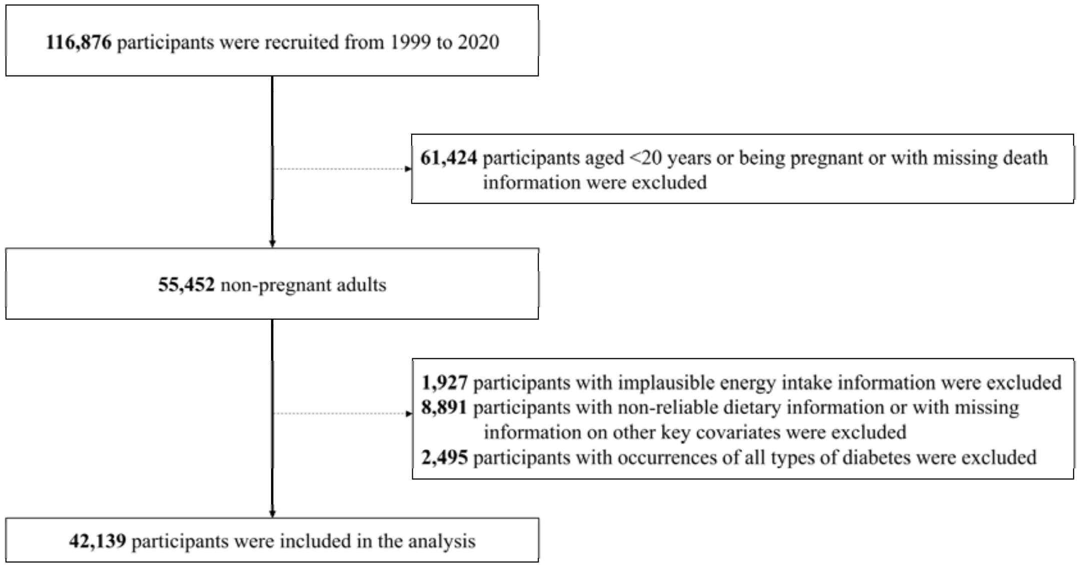

Figure S3 Flow chart for US NHANES

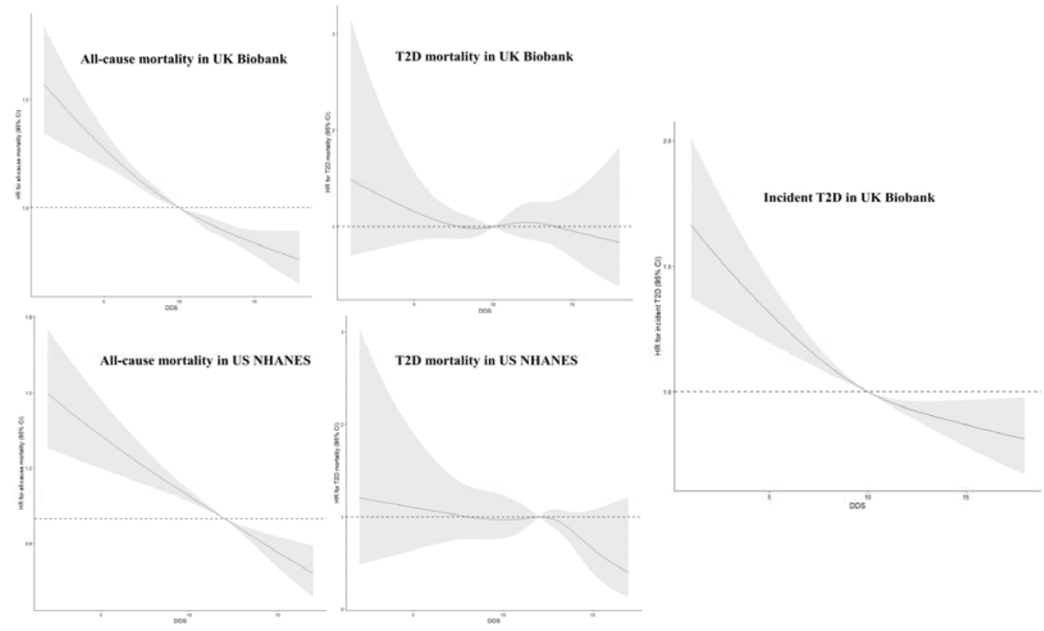

Figure S4 Restricted cubic spline plot of association of DDS with all-cause mortality and incidence and mortality of T2D in UK Biobank and US NHANES. Plots adjusted for sex, age, race, household income, residence (UK Biobank only), alcohol consumption, smoking status, physical activity, family history of diabetes (included in analyses when using incidence or mortality of T2D as outcomes), total calorie intake from diet, dietary supplement, BMI, CVD, cancer, hypertension, and hyperlipemia.

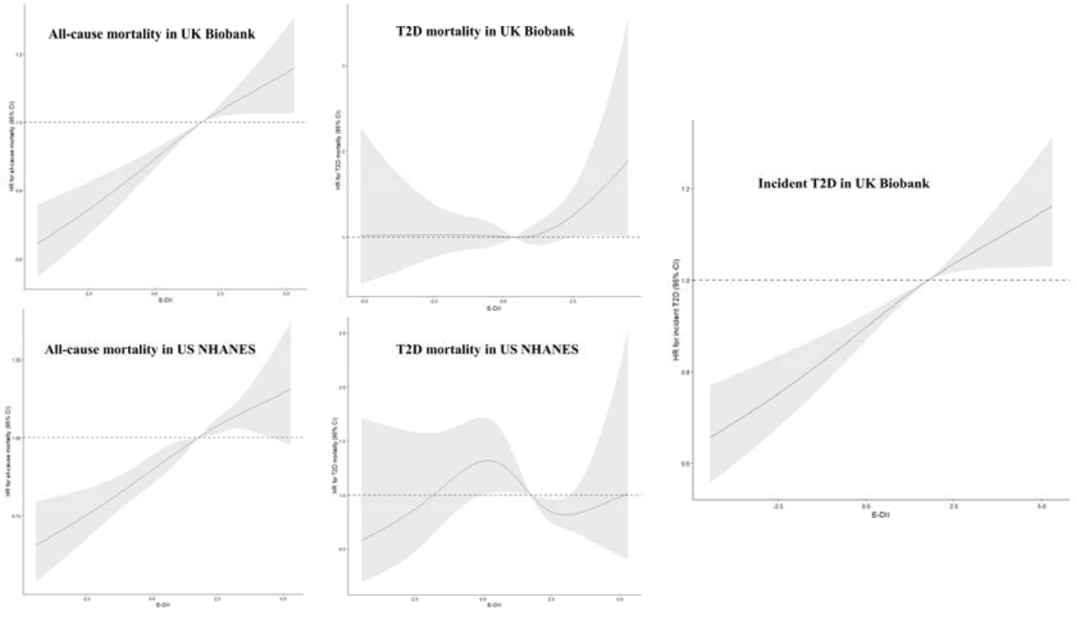

Figure S5 Restricted cubic spline plot of association of E-DII with all-cause mortality and incidence and mortality of T2D in UK Biobank and US NHANES. Plots adjusted for sex, age, race, household income, residence (UK Biobank only), alcohol consumption, smoking status, physical activity, family history of diabetes (included in analyses when using incidence or mortality of T2D as outcomes), total calorie intake from diet, dietary supplement, BMI, CVD, cancer, hypertension, and hyperlipemia.
